# (2,9-Dieth­oxy-1,10-phenanthroline-κ^2^ *N*,*N*′)bis­(thio­cyanato-κ*N*)cobalt(II)

**DOI:** 10.1107/S160053680803496X

**Published:** 2008-10-31

**Authors:** Xian-Fu Zheng, Hui Su, Zhan-Fang Zhou, Chun-Hong Kou, Cao-Yuan Niu

**Affiliations:** a College of Sciences, Henan Agricultural University, Zhengzhou 450002, People’s Republic of China

## Abstract

In the title complex, [Co(NCS)_2_(C_16_H_16_N_2_O_2_)], the Co^II^ ion is coordinated by two N atoms from one 2,9-dieth­oxy-1,10-phenanthroline ligand and two N atoms from two different thio­cyanate ligands in a distorted tetra­hedral environment. The Co—N bonds involving the thio­cyanate ligands are significantly shorter than the other two Co—N bonds. The atoms of one of the eth­oxy groups are essentially coplanar with the phenanthroline ring [N=C—O—C = 178.8 (4)°], while the other eth­oxy group is slightly twisted from the phenanthroline ring plane [N=C—O—C = 167.2 (4)°]. In the crystal structure, there is a weak π–π stacking inter­action between two symmetry-related phenanthroline rings with a centroid–centroid distance of 3.706 (4) Å.

## Related literature

For 1,10-phenanthroline coordination compounds with transition metal atoms as potential strong luminescent materials, see: Majumdera *et al.* (2006 ▶); Bie *et al.* (2006 ▶); Pijper *et al.* (1984 ▶).
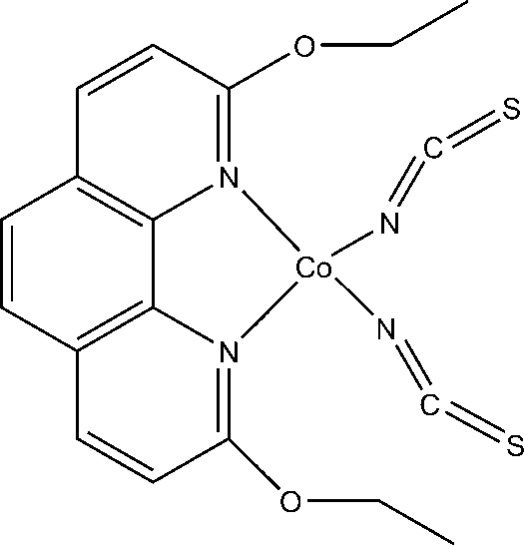


## Experimental

### 

#### Crystal data

[Co(NCS)_2_(C_16_H_16_N_2_O_2_)]
*M* *_r_* = 443.40
Monoclinic, 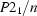

*a* = 8.7072 (16) Å
*b* = 15.625 (3) Å
*c* = 14.828 (3) Å
β = 95.082 (3)°
*V* = 2009.4 (6) Å^3^
*Z* = 4
Mo *K*α radiation
μ = 1.08 mm^−1^
*T* = 291 (2) K
0.34 × 0.20 × 0.10 mm

#### Data collection

Siemens SMART CCD diffractometer
Absorption correction: multi-scan (*SADABS*; Sheldrick, 1996 ▶) *T*_min_ = 0.707, *T*_max_ = 0.899
10517 measured reflections
3726 independent reflections
2904 reflections with *I* > 2σ(*I*)
*R*_int_ = 0.031

#### Refinement

*R*[*F* ^2^ > 2σ(*F* ^2^)] = 0.070
*wR*(*F* ^2^) = 0.239
*S* = 1.07
3726 reflections
246 parameters
H-atom parameters constrained
Δρ_max_ = 1.80 e Å^−3^
Δρ_min_ = −0.49 e Å^−3^

### 

Data collection: *SMART* (Siemens, 1996 ▶); cell refinement: *SAINT* (Siemens, 1994 ▶); data reduction: *SAINT*; program(s) used to solve structure: *SHELXL97* (Sheldrick, 2008 ▶); program(s) used to refine structure: *SHELXL97* (Sheldrick, 2008 ▶); molecular graphics: *DIAMOND* (Brandenburg, 2005 ▶); software used to prepare material for publication: *SHELXTL* (Sheldrick, 2008 ▶).

## Supplementary Material

Crystal structure: contains datablocks I, global. DOI: 10.1107/S160053680803496X/lh2718sup1.cif

Structure factors: contains datablocks I. DOI: 10.1107/S160053680803496X/lh2718Isup2.hkl

Additional supplementary materials:  crystallographic information; 3D view; checkCIF report

## Figures and Tables

**Table:** 

Co1—N3	1.928 (4)
Co1—N4	1.930 (5)
Co1—N1	2.035 (4)
Co1—N2	2.038 (4)

**Table:** 

N3—Co1—N4	109.33 (18)
N3—Co1—N1	116.67 (17)
N4—Co1—N1	119.86 (17)
N3—Co1—N2	114.22 (17)
N4—Co1—N2	113.12 (18)
N1—Co1—N2	81.08 (16)

**Fig. 1.**
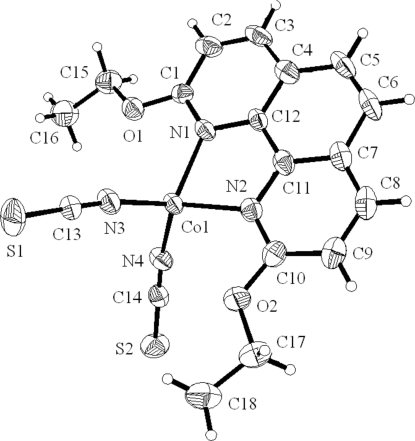
A view of the title complex, showing the atom-labelling scheme. Displacement ellipsoids are drawn at the 30% probability level and H atoms are shown as small spheres of arbitrary radii.

**Fig. 2.**
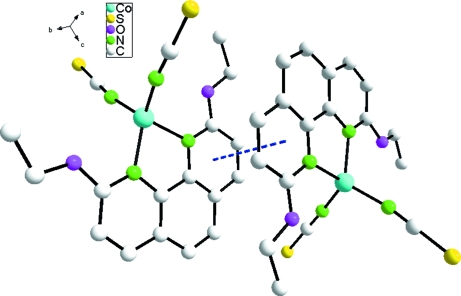
Part of the crystal structure showing intermolecular π–π stacking indicated by dashed lines. All H atoms have been omitted for clarity.

## supplementary crystallographic information

### Comment

Derivatives of 1,10-phenanthroline can be used as multi-dentate ligands. Their coordination compounds with transition metal atoms possess potential as strong luminescent materials (Majumdera *et al.*, 2006; Bie, *et al.*, 2006) and antimycoplasmal activity (Pijper, *et al.*, 1984).

In the title compound the Co^II^ ion is coordinated by two nitrogen atoms from one phenanthroline ring (N1, N2) and two nitrogen atoms from two different thiocyanate ligands (N3, N4) forming a distorted tetrahedral enviroment (Fig. 1). The Co1—N1 and Co1—N2 bond lengths are longer than the Co1—N3 and Co1—N4 bond lengths. The N1—Co1—N2 bond angle of 81.08 (16) ° involving the two phenanthroline nitrogen atoms is the smallest coordination angle (Table 1). All other N—Co1—N bond angles are larger than the ideal 109.5 °. The atoms of one of the ethoxy groups are essentially co-planar with the phenanthroline ring [N2═C10-O2-C17 = 178.8 (4)°] while the other ethoxy group is slightly twisted from the phenanthroline ring plane [N1═C1-O1-C15 = 167.2 (4)°]. In the crystal structure, weak π–π stacking interactions between pairs of symmetry related phenanthroline rings form a centroid-to-centroid distance of 3.706 (4) Å (Fig. 2).

### Experimental

The organic ligand 2,9-diethoxy-1,10-phenanthroline was prepared according to the procedure of literature (Pijper, *et al.*, 1984). The slow evaporation of mixture of the ligand (0.024 g, 0.1 mmol), NH_4_SCN (0.016 g, 0.2 mmol), and Co(ClO_4_)_2_^.^6H_2_O (0.037 g, 0.1 mmol) in 30 ml me thanol afforded blue block single crystals in about 10 days (yield about 67%).

### Refinement

The H atoms were positioned geometrically and refined using a riding model [C—H = 0.93 Å and *U*_iso_(H) = 1.2*U*_eq_(C) for aromatic H atoms; C—H = 0.97 Å and *U*_iso_(H) = 1.2*U*_eq_(C) for methylene H atoms; C—H = 0.96 Å and *U*_iso_(H) = 1.5*U*_eq_(C) for methyl H atoms]. The final difference Fourier map had a highest peak at 0.90 Å from atom O1 and a deepest hole at 0.90 Å from atom S2, but were otherwise featureless.

### Crystal data

**Table:**

[Co(NCS)_2_(C_16_H_16_N_2_O_2_)]	*F*(000) = 908
*M**_r_* = 443.40	*D*_x_ = 1.466 Mg m^−^^3^
Monoclinic, *P*2_1_/*n*	Mo *K*α radiation, λ = 0.71073 Å
Hall symbol: -P 2yn	Cell parameters from 3449 reflections
*a* = 8.7072 (16) Å	θ = 2.6–25.5°
*b* = 15.625 (3) Å	µ = 1.08 mm^−^^1^
*c* = 14.828 (3) Å	*T* = 291 K
β = 95.082 (3)°	Block, blue
*V* = 2009.4 (6) Å^3^	0.34 × 0.20 × 0.10 mm
*Z* = 4	

### Data collection

**Table:**

Siemens SMART CCD diffractometer	3726 independent reflections
Radiation source: fine-focus sealed tube	2904 reflections with *I* > 2σ(*I*)
graphite	*R*_int_ = 0.031
φ and ω scans	θ_max_ = 25.5°, θ_min_ = 2.6°
Absorption correction: multi-scan (SADABS; Sheldrick, 1996)	*h* = −10→10
*T*_min_ = 0.707, *T*_max_ = 0.899	*k* = −18→12
10517 measured reflections	*l* = −17→16

### Refinement

**Table:**

Refinement on *F*^2^	Primary atom site location: structure-invariant direct methods
Least-squares matrix: full	Secondary atom site location: difference Fourier map
*R*[*F*^2^ > 2σ(*F*^2^)] = 0.070	Hydrogen site location: inferred from neighbouring sites
*wR*(*F*^2^) = 0.239	H-atom parameters constrained
*S* = 1.07	*w* = 1/[σ^2^(*F*_o_^2^) + (0.1606*P*)^2^ + 1.5683*P*] where *P* = (*F*_o_^2^ + 2*F*_c_^2^)/3
3726 reflections	(Δ/σ)_max_ < 0.001
246 parameters	Δρ_max_ = 1.80 e Å^−^^3^
0 restraints	Δρ_min_ = −0.49 e Å^−^^3^

### Special details

**Table:**

Geometry. All e.s.d.'s (except the e.s.d. in the dihedral angle between two l.s. planes) are estimated using the full covariance matrix. The cell e.s.d.'s are taken into account individually in the estimation of e.s.d.'s in distances, angles and torsion angles; correlations between e.s.d.'s in cell parameters are only used when they are defined by crystal symmetry. An approximate (isotropic) treatment of cell e.s.d.'s is used for estimating e.s.d.'s involving l.s. planes.
Refinement. Refinement of *F*^2^ against ALL reflections. The weighted *R*-factor *wR* and goodness of fit *S* are based on *F*^2^, conventional *R*-factors *R* are based on *F*, with *F* set to zero for negative *F*^2^. The threshold expression of *F*^2^ > σ(*F*^2^) is used only for calculating *R*-factors(gt) *etc*. and is not relevant to the choice of reflections for refinement. *R*-factors based on *F*^2^ are statistically about twice as large as those based on *F*, and *R*- factors based on ALL data will be even larger.

### Fractional atomic coordinates and isotropic or equivalent isotropic displacement parameters (Å^2^)

**Table:**

	*x*	*y*	*z*	*U*_iso_*/*U*_eq_	
Co1	0.90632 (7)	0.18324 (4)	0.31994 (4)	0.0446 (3)	
S1	0.6802 (3)	0.18997 (12)	0.02186 (11)	0.0835 (6)	
S2	1.4386 (2)	0.18412 (11)	0.30008 (16)	0.0841 (6)	
O1	0.8363 (4)	0.3691 (2)	0.3802 (2)	0.0599 (9)	
O2	0.9610 (5)	−0.0115 (2)	0.2896 (3)	0.0668 (10)	
N1	0.8007 (4)	0.2359 (3)	0.4237 (2)	0.0474 (9)	
N2	0.8523 (5)	0.0737 (3)	0.3847 (3)	0.0508 (10)	
N3	0.8028 (5)	0.1958 (3)	0.2003 (3)	0.0577 (11)	
N4	1.1253 (5)	0.1983 (3)	0.3143 (3)	0.0571 (11)	
C1	0.7782 (5)	0.3180 (3)	0.4418 (3)	0.0461 (11)	
C2	0.7004 (7)	0.3438 (4)	0.5164 (4)	0.0658 (15)	
H2	0.6861	0.4017	0.5275	0.079*	
C3	0.6473 (7)	0.2855 (5)	0.5710 (4)	0.0685 (16)	
H3	0.5963	0.3032	0.6203	0.082*	
C4	0.6667 (6)	0.1969 (4)	0.5557 (4)	0.0601 (14)	
C5	0.6155 (7)	0.1293 (5)	0.6092 (4)	0.0719 (17)	
H5	0.5611	0.1424	0.6586	0.086*	
C6	0.6434 (7)	0.0464 (5)	0.5905 (4)	0.0741 (18)	
H6	0.6090	0.0036	0.6274	0.089*	
C7	0.7254 (6)	0.0239 (4)	0.5145 (4)	0.0593 (13)	
C8	0.7608 (7)	−0.0601 (4)	0.4899 (4)	0.0698 (16)	
H8	0.7302	−0.1055	0.5247	0.084*	
C9	0.8390 (7)	−0.0767 (4)	0.4160 (4)	0.0680 (15)	
H9	0.8624	−0.1326	0.4004	0.082*	
C10	0.8837 (6)	−0.0064 (3)	0.3637 (4)	0.0556 (12)	
C11	0.7747 (5)	0.0888 (3)	0.4597 (3)	0.0508 (12)	
C12	0.7455 (5)	0.1759 (3)	0.4801 (3)	0.0455 (11)	
C13	0.7502 (6)	0.1927 (3)	0.1260 (4)	0.0503 (12)	
C14	1.2557 (6)	0.1921 (3)	0.3084 (3)	0.0512 (12)	
C15	0.8523 (7)	0.4599 (4)	0.3978 (4)	0.0715 (16)	
H15A	0.9004	0.4697	0.4585	0.086*	
H15B	0.7522	0.4875	0.3922	0.086*	
C16	0.9501 (8)	0.4942 (4)	0.3297 (5)	0.090 (2)	
H16A	1.0500	0.4678	0.3375	0.135*	
H16B	0.9608	0.5550	0.3374	0.135*	
H16C	0.9032	0.4821	0.2701	0.135*	
C17	1.0080 (7)	−0.0937 (4)	0.2575 (5)	0.0732 (16)	
H17A	0.9188	−0.1287	0.2388	0.088*	
H17B	1.0713	−0.1237	0.3045	0.088*	
C18	1.0986 (8)	−0.0743 (5)	0.1784 (5)	0.092 (2)	
H18A	1.0366	−0.0409	0.1348	0.139*	
H18B	1.1274	−0.1269	0.1510	0.139*	
H18C	1.1897	−0.0428	0.1989	0.139*	

### Atomic displacement parameters (Å^2^)

**Table:**

	*U*^11^	*U*^22^	*U*^33^	*U*^12^	*U*^13^	*U*^23^
Co1	0.0453 (4)	0.0551 (5)	0.0342 (4)	−0.0015 (3)	0.0081 (3)	0.0025 (3)
S1	0.1125 (15)	0.0887 (12)	0.0451 (9)	−0.0064 (9)	−0.0165 (9)	0.0053 (7)
S2	0.0513 (9)	0.0844 (12)	0.1190 (16)	0.0024 (7)	0.0205 (9)	0.0091 (10)
O1	0.071 (2)	0.058 (2)	0.052 (2)	0.0013 (17)	0.0125 (17)	−0.0004 (17)
O2	0.080 (3)	0.053 (2)	0.069 (2)	0.0074 (18)	0.017 (2)	−0.0040 (18)
N1	0.0404 (19)	0.068 (3)	0.0341 (19)	−0.0015 (17)	0.0057 (15)	0.0015 (18)
N2	0.049 (2)	0.059 (2)	0.044 (2)	−0.0027 (18)	0.0031 (16)	0.0038 (19)
N3	0.052 (2)	0.079 (3)	0.042 (2)	−0.003 (2)	0.0059 (19)	0.004 (2)
N4	0.047 (2)	0.079 (3)	0.047 (2)	−0.005 (2)	0.0087 (18)	−0.001 (2)
C1	0.042 (2)	0.051 (3)	0.045 (3)	0.0039 (18)	0.0017 (19)	−0.005 (2)
C2	0.062 (3)	0.082 (4)	0.054 (3)	0.011 (3)	0.011 (3)	−0.017 (3)
C3	0.058 (3)	0.108 (5)	0.042 (3)	0.008 (3)	0.017 (2)	−0.014 (3)
C4	0.044 (3)	0.096 (4)	0.039 (3)	0.000 (2)	−0.001 (2)	−0.001 (3)
C5	0.060 (3)	0.119 (6)	0.039 (3)	−0.013 (3)	0.016 (2)	0.008 (3)
C6	0.067 (4)	0.110 (5)	0.047 (3)	−0.022 (3)	0.013 (3)	0.020 (3)
C7	0.053 (3)	0.076 (4)	0.048 (3)	−0.016 (3)	−0.001 (2)	0.013 (3)
C8	0.068 (4)	0.076 (4)	0.063 (4)	−0.016 (3)	−0.007 (3)	0.026 (3)
C9	0.070 (4)	0.059 (3)	0.072 (4)	−0.008 (3)	−0.007 (3)	0.012 (3)
C10	0.053 (3)	0.056 (3)	0.055 (3)	0.001 (2)	−0.006 (2)	0.002 (2)
C11	0.044 (2)	0.070 (3)	0.037 (2)	−0.007 (2)	−0.0017 (18)	0.008 (2)
C12	0.037 (2)	0.064 (3)	0.035 (2)	−0.0055 (19)	0.0021 (18)	0.008 (2)
C13	0.055 (3)	0.052 (3)	0.044 (3)	0.001 (2)	0.007 (2)	0.005 (2)
C14	0.060 (3)	0.051 (3)	0.043 (3)	−0.005 (2)	0.007 (2)	−0.001 (2)
C15	0.072 (4)	0.064 (4)	0.079 (4)	0.000 (3)	0.008 (3)	−0.009 (3)
C16	0.090 (5)	0.065 (4)	0.120 (6)	−0.007 (4)	0.033 (4)	0.009 (4)
C17	0.069 (4)	0.062 (3)	0.088 (4)	0.007 (3)	−0.001 (3)	−0.016 (3)
C18	0.090 (5)	0.084 (5)	0.106 (6)	0.006 (4)	0.026 (4)	−0.022 (4)

### Geometric parameters (Å, °)

**Table:**

Co1—N3	1.928 (4)	C5—C6	1.352 (9)
Co1—N4	1.930 (5)	C5—H5	0.9300
Co1—N1	2.035 (4)	C6—C7	1.431 (8)
Co1—N2	2.038 (4)	C6—H6	0.9300
S1—C13	1.609 (5)	C7—C11	1.391 (7)
S2—C14	1.613 (6)	C7—C8	1.403 (9)
O1—C1	1.345 (6)	C8—C9	1.365 (9)
O1—C15	1.447 (7)	C8—H8	0.9300
O2—C10	1.341 (7)	C9—C10	1.419 (8)
O2—C17	1.441 (7)	C9—H9	0.9300
N1—C1	1.329 (6)	C11—C12	1.422 (7)
N1—C12	1.371 (6)	C15—C16	1.478 (8)
N2—C10	1.324 (7)	C15—H15A	0.9700
N2—C11	1.372 (6)	C15—H15B	0.9700
N3—C13	1.156 (7)	C16—H16A	0.9600
N4—C14	1.151 (7)	C16—H16B	0.9600
C1—C2	1.406 (7)	C16—H16C	0.9600
C2—C3	1.330 (9)	C17—C18	1.501 (9)
C2—H2	0.9300	C17—H17A	0.9700
C3—C4	1.415 (9)	C17—H17B	0.9700
C3—H3	0.9300	C18—H18A	0.9600
C4—C12	1.404 (7)	C18—H18B	0.9600
C4—C5	1.417 (9)	C18—H18C	0.9600
			
N3—Co1—N4	109.33 (18)	C7—C8—H8	119.2
N3—Co1—N1	116.67 (17)	C8—C9—C10	118.2 (6)
N4—Co1—N1	119.86 (17)	C8—C9—H9	120.9
N3—Co1—N2	114.22 (17)	C10—C9—H9	120.9
N4—Co1—N2	113.12 (18)	N2—C10—O2	112.2 (4)
N1—Co1—N2	81.08 (16)	N2—C10—C9	122.1 (5)
C1—O1—C15	119.7 (4)	O2—C10—C9	125.7 (5)
C10—O2—C17	120.1 (4)	N2—C11—C7	123.2 (5)
C1—N1—C12	118.1 (4)	N2—C11—C12	116.5 (4)
C1—N1—Co1	128.9 (3)	C7—C11—C12	120.3 (5)
C12—N1—Co1	113.0 (3)	N1—C12—C4	123.3 (5)
C10—N2—C11	118.7 (4)	N1—C12—C11	116.5 (4)
C10—N2—Co1	128.5 (3)	C4—C12—C11	120.1 (4)
C11—N2—Co1	112.8 (3)	N3—C13—S1	178.6 (5)
C13—N3—Co1	170.6 (4)	N4—C14—S2	179.6 (5)
C14—N4—Co1	168.0 (4)	O1—C15—C16	106.5 (5)
N1—C1—O1	111.4 (4)	O1—C15—H15A	110.4
N1—C1—C2	121.7 (5)	C16—C15—H15A	110.4
O1—C1—C2	126.9 (5)	O1—C15—H15B	110.4
C3—C2—C1	120.0 (6)	C16—C15—H15B	110.4
C3—C2—H2	120.0	H15A—C15—H15B	108.6
C1—C2—H2	120.0	C15—C16—H16A	109.5
C2—C3—C4	121.3 (5)	C15—C16—H16B	109.5
C2—C3—H3	119.3	H16A—C16—H16B	109.5
C4—C3—H3	119.3	C15—C16—H16C	109.5
C12—C4—C3	115.5 (5)	H16A—C16—H16C	109.5
C12—C4—C5	118.3 (6)	H16B—C16—H16C	109.5
C3—C4—C5	126.2 (6)	O2—C17—C18	105.2 (5)
C6—C5—C4	121.9 (5)	O2—C17—H17A	110.7
C6—C5—H5	119.1	C18—C17—H17A	110.7
C4—C5—H5	119.1	O2—C17—H17B	110.7
C5—C6—C7	120.6 (5)	C18—C17—H17B	110.7
C5—C6—H6	119.7	H17A—C17—H17B	108.8
C7—C6—H6	119.7	C17—C18—H18A	109.5
C11—C7—C8	116.4 (5)	C17—C18—H18B	109.5
C11—C7—C6	118.8 (6)	H18A—C18—H18B	109.5
C8—C7—C6	124.8 (5)	C17—C18—H18C	109.5
C9—C8—C7	121.5 (5)	H18A—C18—H18C	109.5
C9—C8—H8	119.2	H18B—C18—H18C	109.5
			
N3—Co1—N1—C1	−68.1 (4)	C6—C7—C8—C9	179.7 (5)
N4—Co1—N1—C1	67.6 (4)	C7—C8—C9—C10	−0.3 (8)
N2—Co1—N1—C1	179.2 (4)	C11—N2—C10—O2	−179.4 (4)
N3—Co1—N1—C12	111.0 (3)	Co1—N2—C10—O2	−0.1 (6)
N4—Co1—N1—C12	−113.3 (3)	C11—N2—C10—C9	0.3 (7)
N2—Co1—N1—C12	−1.7 (3)	Co1—N2—C10—C9	179.6 (4)
N3—Co1—N2—C10	66.4 (4)	C17—O2—C10—N2	178.8 (4)
N4—Co1—N2—C10	−59.6 (5)	C17—O2—C10—C9	−0.9 (8)
N1—Co1—N2—C10	−178.3 (4)	C8—C9—C10—N2	0.3 (8)
N3—Co1—N2—C11	−114.3 (3)	C8—C9—C10—O2	179.9 (5)
N4—Co1—N2—C11	119.8 (3)	C10—N2—C11—C7	−0.8 (7)
N1—Co1—N2—C11	1.0 (3)	Co1—N2—C11—C7	179.8 (4)
N3—Co1—N4—C14	−90 (2)	C10—N2—C11—C12	179.2 (4)
N1—Co1—N4—C14	132 (2)	Co1—N2—C11—C12	−0.2 (5)
N2—Co1—N4—C14	39 (2)	C8—C7—C11—N2	0.7 (7)
C12—N1—C1—O1	−179.1 (4)	C6—C7—C11—N2	−179.1 (4)
Co1—N1—C1—O1	0.0 (6)	C8—C7—C11—C12	−179.3 (5)
C12—N1—C1—C2	0.0 (7)	C6—C7—C11—C12	0.8 (7)
Co1—N1—C1—C2	179.1 (4)	C1—N1—C12—C4	0.0 (7)
C15—O1—C1—N1	−167.2 (4)	Co1—N1—C12—C4	−179.2 (4)
C15—O1—C1—C2	13.7 (8)	C1—N1—C12—C11	−178.7 (4)
N1—C1—C2—C3	0.1 (8)	Co1—N1—C12—C11	2.1 (5)
O1—C1—C2—C3	179.0 (5)	C3—C4—C12—N1	−0.2 (7)
C1—C2—C3—C4	−0.3 (9)	C5—C4—C12—N1	−179.8 (5)
C2—C3—C4—C12	0.3 (8)	C3—C4—C12—C11	178.4 (5)
C2—C3—C4—C5	179.9 (5)	C5—C4—C12—C11	−1.1 (7)
C12—C4—C5—C6	1.4 (8)	N2—C11—C12—N1	−1.3 (6)
C3—C4—C5—C6	−178.1 (6)	C7—C11—C12—N1	178.8 (4)
C4—C5—C6—C7	−0.6 (9)	N2—C11—C12—C4	180.0 (4)
C5—C6—C7—C11	−0.6 (8)	C7—C11—C12—C4	0.0 (7)
C5—C6—C7—C8	179.6 (6)	C1—O1—C15—C16	167.3 (5)
C11—C7—C8—C9	−0.1 (8)	C10—O2—C17—C18	−176.5 (5)
